# Screening of NIAS World Rice Core Collection for Seeds with Long Longevity as Useful Potential Breeding Materials Focusing on the Stability of Embryonic RNAs

**DOI:** 10.3390/plants13131869

**Published:** 2024-07-06

**Authors:** Kalimullah Saighani, Megumi Kashiwagi, Safiullah Habibi, Craig G. Simpson, Tetsuya Yamada, Motoki Kanekatsu

**Affiliations:** 1 School of Biology, Biomedical Sciences Research Complex, University of St. Andrews, Fife KY16 9TS, UK; 2 United Graduate School of Agricultural Science, Tokyo University of Agriculture and Technology, Tokyo 183-8509, Japan; megumi.kashiwagi@gmail.com (M.K.); teyamada@cc.tuat.ac.jp (T.Y.); kanekatu@cc.tuat.ac.jp (M.K.); 3 Faculty of Agriculture, Tokyo University of Agriculture and Technology, Saiwai-Cho 3-5-8, Tokyo 183-8509, Japan; safiullahhabibi23@gmail.com; 4 Cell and Molecular Sciences, The James Hutton Institute, Dundee DD2 5DA, UK; craig.simpson@hutton.ac.uk

**Keywords:** world rice (*Oryza sativa* L.) core collection, seed longevity, embryonic RNA stability, RNA integrity number (RIN), controlled deterioration treatment (CDT), breeding materials

## Abstract

Seed longevity is a crucial trait for the seed industry and genetic resource preservation. To develop excellent cultivars with extended seed lifespans, it is important to understand the mechanism of keeping seed germinability long term and to find useful genetic resources as prospective breeding materials. This study was conducted to identify the best cultivars with a high and stable seed longevity trait in the germplasm of rice (*Oryza sativa* L.) and to analyze the correlation between seed longevity and embryonic RNA integrity. Seeds from 69 cultivars of the world rice core collection selected by the NIAS in Japan were harvested in different years and subjected to long-term storage or controlled deterioration treatment (CDT). The long-term storage (4 °C, RH under 35%, 10 years) was performed on seeds harvested in 2010 and 2013. The seeds harvested in 2016 and 2019 were used for CDT (36 °C, RH of 80%, 40 days). Seed longevity and embryonic RNA integrity were estimated by a decrease in the germination percentage and RNA integrity number (RIN) after long-term storage or CDT. The RIN value was obtained by the electrophoresis of the total RNA extracted from the seed embryos. Seeds of “Vandaran (*indica*)”, “Tupa 729 (*japonica*)”, and “Badari Dhan (*indica*)” consistently showed higher seed longevity and embryonic RNA integrity both under long-term storage and CDT conditions regardless of the harvest year. A strong correlation (R^2^ = 0.93) was observed between the germination percentages and RIN values of the seeds after the long-term storage or CDT among nine cultivars selected based on differences in their seed longevity. The study findings revealed the relationship between rice seed longevity and embryo RNA stability and suggested potential breeding materials including both *japonica* and *indica* cultivars for improving rice seed longevity.

## 1. Introduction

Seed longevity is the period during which a seed remains viable during storage. The germinability of seeds decreases progressively over time due to the aging process, which then leads to weak seedling growth and poor crop productivity [1,2,3]. High-viability seeds produce robust seedlings and can ultimately ensure a high yield. It is, therefore, an important trait for the seed industry and the preservation of genetic resources [4,5,6,7]. There are various crops, such as green onions and carrots, with a shorter lifespan of seeds [8], while species like sacred lotus (*Nelumbo nucifera*) and *Phoenix dactylifera* exhibit significantly longer seed longevity [9,10]. However, the mechanism to maintain the viability of seeds over the long term is not yet well understood. The seed’s longevity is partially affected by genetic factors but is also influenced by the environmental conditions experienced by the mother plant during seed maturation and the conditions imposed during the post-harvest and storage periods [1,11,12]. Hence, this valuable agronomic trait can be improved by breeding methods. To create excellent cultivars with seeds expanding in longevity by breeding, it is important to understand the mechanism of keeping seed germinability long term and to find useful genetic resources as prospective breeding materials.

It is generally accepted that seed longevity widely varies according to the cultivars of rice [13,14,15,16,17], rye, wheat, and triticale [18], and this trait may be improved by breeding among other species [19,20,21,22]. Screening multiple cultivars for seed longevity will identify useful cultivars as potential breeding materials with long and stable seed longevity traits. There are about 37,000 rice cultivars cultivated across the world [23]. The sheer number of rice cultivars makes it unfeasible to study and screen this large scale of rice varieties for the target trait. To address this challenge, a useful collection of rice can be utilized to determine cultivars with desirable target traits. The NIAS world rice core collection of rice germplasms has been established by the National Agriculture and Food Research Organization (NARO) in Japan [23,24]. This collection is a set of world rice cultivars called RDRS (rice diversity research set of germplasms), which was developed from roughly 300 accessions of rice selected according to their origin information and determined 69 cultivars based on a restriction fragment length polymorphism (RFLP), which covers about 90% of allelic diversity in rice varieties cultivated across the world. 

RDRS varieties are classified into three genotype groups based on RFLP data, with “Nipponbare” and “Kasalath” as reference varieties [25,26,27]. Group A is a “*japonica (j)* group” with the “Nipponbare” allele, while Group B is an “*india-1*” group with aus varieties from India, Bangladesh, Bhutan, Nepal, and Sri Lanka, and Group C is an “*indica-2*” group with *indica* varieties from Madagascar to China. The “Kasalath” allele is predominant in Groups B and C, with a higher ratio in Group B. The RDRS collection has been used for the evaluation of several agricultural traits, such as heading date, seed cadmium concentration, and seed dormancy [28,29,30,31,32,33]. Therefore, this suggests that the RDRS, also known as the world rice core collection, is a powerful tool for investigating and identifying potential breeding materials in rice. For example, useful breeding materials with heat-stress tolerance in seeds under hot water disinfection were discovered by using this core collection of RDRS [34]. According to Kashiwagi’s report, the seeds of two *japonica* cultivars, “Rexmont” and “Tupa 729”, and an *indica* cultivar, “Badari Dhan”, had shown extremely high heat-stress resistance, which can be used as useful genetic resources to identify the gene(s) inducing heat-stress tolerance during the hot water disinfection process. It has been proposed that agricultural traits in seeds are greatly influenced by the cultivation environment, and seeds cultivated in different fields and years are required for experiments to evaluate their traits [34]. 

It has been shown that seed longevity is highly associated with the stability of the total RNA content and integrity in the dry seeds of numerous crops, including peas [35], sunflower [36], Nicotiana species [37], garden pea [38], and mung bean [39]. An effective technique for examining the integrity of RNA is total RNA electrophoresis, which is followed by the computation of the RNA integrity number (RIN) that assesses the RNA fragment size distribution [1,40]. In an electrophoretic examination of total RNA from plant tissues, the most abundant RNAs in plant cells, the 25S and 18S rRNAs, can easily be identified as the primary bands whereas smears or extra bands may be seen when RNAs have begun to degrade. An Agilent 2100 Bioanalyzer software (B.02.11) calculates RIN values based on the ratio and height of peaks corresponding to 25S and 18S rRNA bands and the presence of additional bands in inter-peak regions. The RIN ranges from 1 (fully degraded RNA) to 10 (intact RNA) and is widely used to evaluate RNA integrity in gene expression analysis, such as RNA-seq [1,40]. It has been reported [41,42] that the RIN value is significantly associated with the germination potential in the dry seeds of several crops, such as soybean, pea, carrot, red clover, lettuce, onion, safflower, sesame, and sorghum. We have also found a significantly positive correlation between RIN values and longevity in the seeds of three *japonica* rice cultivars, “Nipponbare”, “Sasanishiki”, and “Koshihikari”, either under CDT or long-term storage at 4 °C [1]. Thus, maintaining the integrity of embryonic RNA is crucial for preserving seed germinability, and the RIN value can be used as a standard index to assess the longevity potential of seeds. Multiple rice cultivars, such as *japonica, indica*, and *aus,* are grown all over the tropical regions of the world and this index needs to be tested across many rice cultivars.

This study sought to identify the rice cultivar(s) with a long and stable seed longevity trait to be used as potential breeding materials, through screening performed on the germinability of rice seeds after “CDT” or “long-time ageing at 4 °C” using the NIAS world rice core collection. Thus, the correlation between the seed longevity and RIN values was analyzed using various *cultivars* of the NIAS world rice core collection. 

## 2. Results

### 2.1. Evaluation of Longevity in Seeds of Cultivars Belonging to the NIAS World Rice Core Collection under Controlled Deterioration Treatment (CDT)

**Figure 1:**
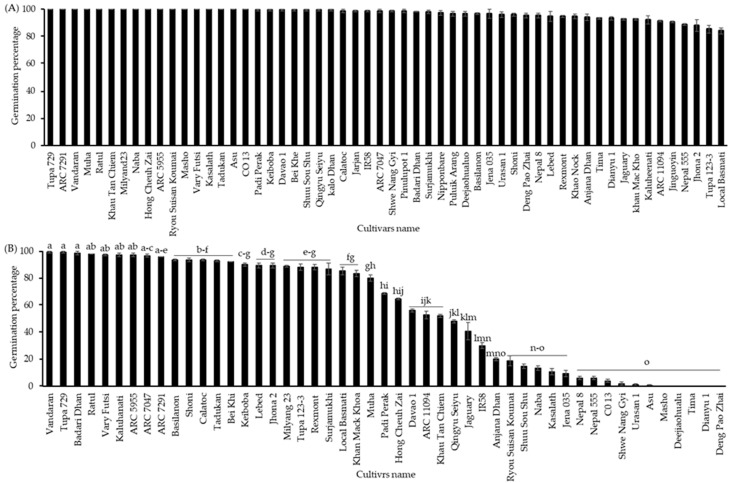
Seed germination percentages of the NIAS world rice core collection was cultivated and harvested in 2016 (**A**) without CDT and (**B**) with CDT (36 °C and 80% relative humidity) for 40 days. The seeds of all cultivars were incubated in Petri dishes (90 × 15 mm) with water at 28 °C for 10 days under darkness. Black bar represents the NIAS world rice core collection germination percentages. Different letters indicate significant differences among cultivars according to the Tukey–Kramer multiple range test at a 5% level.

Firstly, the germinability of the seeds from 49 cultivars of the NIAS world rice core collection harvested in 2016 that were stored at 4 °C for one year was analyzed both with and without CDT. The seeds of almost all cultivars germinated approximately 100% without CDT, but a few cultivars (“Local Basmati”, “Tupa 123-3”, “Jhona 2”, and “Nepal 555”) showed relatively low germination percentages in the range of 85% to 89% (Figure 1A). Seeds of all cultivars were exposed to a condition of CDT with a high temperature and relative humidity (36 °C, 80% RH) for 40 days. Screening of the world rice core collection after 40 days of treatment with CDT clearly showed significant differences in germinability among the cultivars, ranging from 0 to 100%. Among all, the germination percentage of the cultivars “Vandaran”, “Tupa 729”, “Badari Dhan”, “Ratul”, “Vary Futsi”, “Kaluheenati”, “ARC5955”, “ARC7047”, and “ARC7291” remained about 100%, while some other 35 cultivars displayed different germination percentages from 2 to 93%, and 5 other cultivars, “Masho”, “Deejiaohulua”, “Tima”, “Dianyu1”, and “Deng Pao Zhai”, completely lost their germinability (Figure 1B).

**Figure 2:**
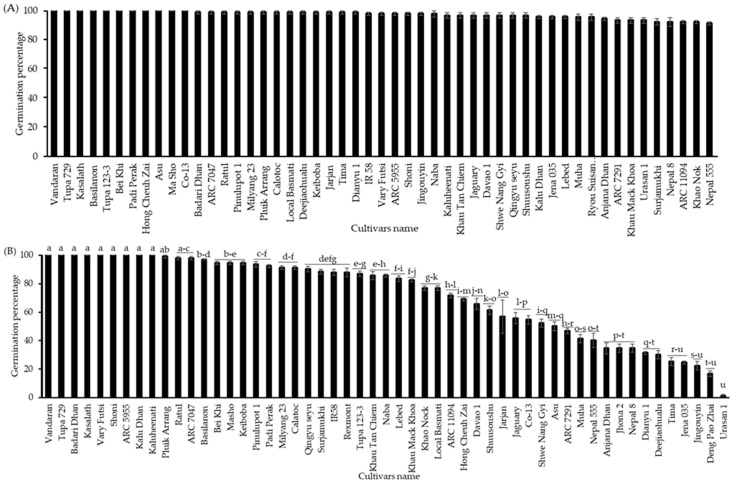
Seed germination percentages of the NIAS world rice core collection was cultivated and harvested in 2019 (**A**) without CDT and (**B**) with CDT at 36 °C and 80% relative humidity for 40 days. The seeds of all cultivars were incubated in Petri dishes (90 × 15 mm) with water at 28 °C for 10 days under darkness. Black bar represents the NIAS world rice core collection germination percentages. Different letters indicate significant differences among cultivars according to the Tukey–Kramer multiple range test at a 5% level.

In addition, to confirm the annual variation in the cultivar differences in the germination percentage of seeds with CDT, the seeds from 53 cultivars grown in 2019 were tested for germination with or without CDT and after storage at 4 °C for 6 months. Before being exposed to CDT, all cultivars germinated nearly 100%, indicating that the seeds of all cultivars are viable and non-dormant (Figure 2A). Meanwhile, after 40 days of being subjected to CDT, significant differences were observed in seed germinability, ranging from 1 to 100%. The top 15 cultivars germinated nearly 100%, and the germinability of others varied from 1 to 94% (Figure 2B). The seeds of the top group of cultivars (“Vandaran”, “Tupa 729”, and “Badari Dhan”) were germinated at 100%, the same as those harvested in 2016. Conversely, the germination percentages of all other cultivars (including the middle and low group cultivars) were significantly variable between the 2016 and 2019 harvest years. The selection of the top, middle, and low cultivars of each class was based on the similarity of their germination percentages after the CDT treatment between the 2016 and 2019 harvest years.

### 2.2. Evaluation of Longevity in Aged Seeds of the NIAS World Rice Core Collection without CDT

**Figure 3:**
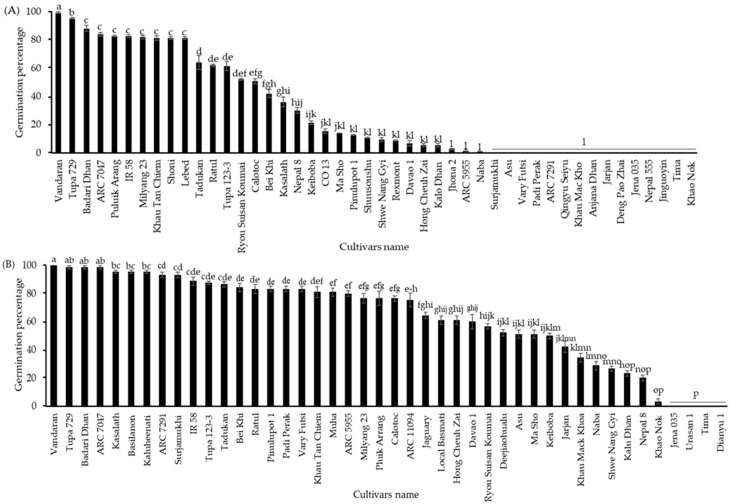
Germination percentage of seeds of NIAS world rice core collection cultivated (**A**) in 2010 and stored at 4 °C for 10 years and (**B**) in 2013 and stored at 4 °C for 7 years. The seeds of all cultivars were incubated in Petri dishes (90 × 15 mm) with water at 28 °C for 10 days under darkness. Black bars represent the NIAS world rice core collection germination percentages. Different letters indicate significant differences among cultivars according to the Tukey–Kramer multiple range test at a 5% level.

To check the cultivar differences in germination percentages between seeds with CDT and aged seeds, the germination assay was performed on seeds grown in 2010 and stored at 4 °C for 10 years. Significant cultivar differences were observed in seed germination percentages, ranging from 0 to approximately 100%. Among them, “Vandaran” showed the highest germination percentage, at about 100%. The seeds of “Tupa 729” and “Badari Dhan” also had high germinability at 95% and 88%, respectively. The germination percentage of 27 cultivars varied from 3 to 80%, and 16 other cultivars completely lost their germinability (Figure 3A). “Vandaran” consistently displayed the highest germination percentage (100%) either under CDT and between the different harvest years of 2016 and 2019. However, the germinability of all other cultivars was significantly variable depending on the aging conditions or between the distinct harvest years. To confirm the annual variation in the cultivar differences in the germination percentage of aged seeds, the seeds harvested in 2013 and aged at 4 °C for 7 years were used for the next germination test, and a significant difference was identified among the cultivars in the performance of seed germination ability. Significant differences were found in the germinability of seeds, ranging from 0 to 100%. Among all, “Vandaran” displayed the highest germination percentage of 100%; seeds from three cultivars such as “Tupa 729”, “Badari Dhan”, and “ARC7047” germinated 99%, the germinability of other cultivars differed from 3 to 95%; and four others, “Jena 035”, “Urasan 1”, “Tima”, and “Dianyu 1” completely lost their germinability (Figure 3B). The substantial decrease in the germination percentage of rice varieties after 10 years of storage, compared to 7 years of storage, might be due to the natural seed aging process and the cultivars’ genetic potential viability. The weather conditions during the cultivation and harvesting of the mother plant may also have affected this decrease.

### 2.3. RNA Integrity in the Embryos of Seeds from the NIAS World Rice Core Collection under CDT and Long-Term Aging at 4 °C

In order to conduct a comparative investigation of the RNA integrity in the embryos of the seeds, three cultivars from the top-, three cultivars from the middle-, and three cultivars from the low-class varieties were selected based on the similarity of their germination percentages after the CDT treatment and between different harvested years. Among the top-class varieties, “Vandaran” germinated 100% in both the 2010 and 2013 harvests, while “Tupa 729” and “Badari Dhan” showed a significantly higher germination percentage (99%) in 2013 compared to the 2010 harvest. The germinability of the middle-class cultivars (“Hong Cheuh Zai”, “Local Basmati”, and “Davao 1”) was significantly higher in the aged seeds harvested in 2013 compared to the data from the 2010 harvest, while the low-class cultivars (“Jena 035”, “Dianyu 1”, and “Urasan 1”) completely lost germinability in both the 2010 and 2013 harvests. For these cultivars, the RNA integrity in the embryos before and after CDT of the seeds harvested in 2019 and stored at 4 °C for 6 months, and in the embryos of the aged seeds harvested in 2013 and stored for 7 years at 4 °C, was compared by electrophoresis.

**Figure 4:**
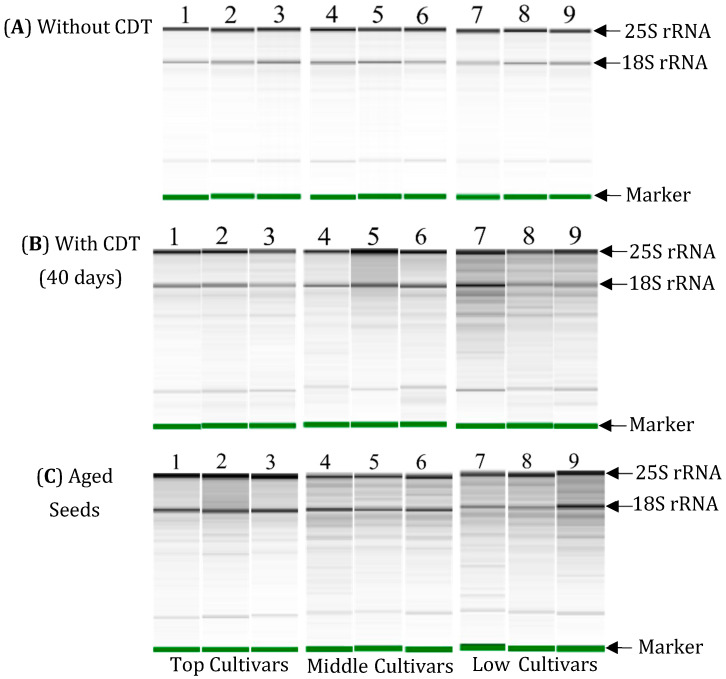
Electrophoresis patterns of total RNA extracted from embryos of “Top, Middle, and Low cultivars” in NIAS world rice core collection harvested in 2019 and 2013, which were stored at 4 °C for 6 months and 7 years, respectively. The total RNAs from embryos (n = 20) without (**A**) or with CDT (**B**) for 40 days, and aged seeds at 4 °C for 7 years (**C**) were analyzed. Lane 1: Vandaran, Lane 2: Tupa729, Lane 3: Badari Dhan, Lane 4: Local Basmati, Lane 5: Hong Cheuh Zai, Lane 6: Davao 1, Lane 7: Urasan 1, Lane 8: Jena 035; Lane 9: Dianyu 1. “Marker” indicates an RNA marker that is added to the samples to align the RNA bands between the different samples.

The results of the electrophoresis show that 25S and 18S rRNA were detected clearly as major bands and there was no RNA degradation in the total RNAs isolated from the embryos before CDT in the seeds of all the selected cultivars (Figure 4A). After CDT for 40 days, there was more evidence of RNA degradation particularly in the “middle” and “low cultivar” selections. In the embryos of the “top cultivars”, RNA remained largely intact with only limited degradation after CDT (Figure 4B). Similarly, in the electrophoresis of total RNAs isolated from the embryos of the aged seeds, some degradation was detected under the position of the 25S and 18S rRNA in the “top cultivars”. However, there was more evidence of RNA degradation in the seeds of the “middle cultivars” and “low cultivars”, because many bands of RNA fragments with a smaller molecular size than rRNA were detected in the extracts from the embryos of these cultivars (Figure 4C).

The results of the electrophoretic analysis shown in Figure 4 were used to calculate the RIN values using the Agilent Bioanalyzer software. The mean RIN values for the total RNA isolated from the embryos of the seeds before CDT (fresh seed) were 8.5 in all nine cultivars used (Table 1). After 40 days of CDT, the mean RIN value of the three “top cultivars” was 8.4, but the RIN values decreased in the “middle cultivars” to 7.7, 7.5, and 7.3, and more markedly decreased in the “low cultivars” to RIN values less than 6. In all nine cultivars investigated, the mean RIN value of the aged seeds was lower than that of the fresh seeds. The germination percentage decreased in the mean RIN value of the aged seeds relative to that of the fresh seeds to 5.9% in the “top cultivars”, 21.2–22.4% in the “middle cultivars”, and 32.9–34.1% in the “low cultivars”. These results show that the RNA stability is highest in the embryos of the seeds in “Vandaran”, “Tupa 729”, and “Badari Dhan” (“top cultivars”) after CDT and natural aging.

### 2.4. Relationship between Germinability and RIN Values

**Figure 5:**
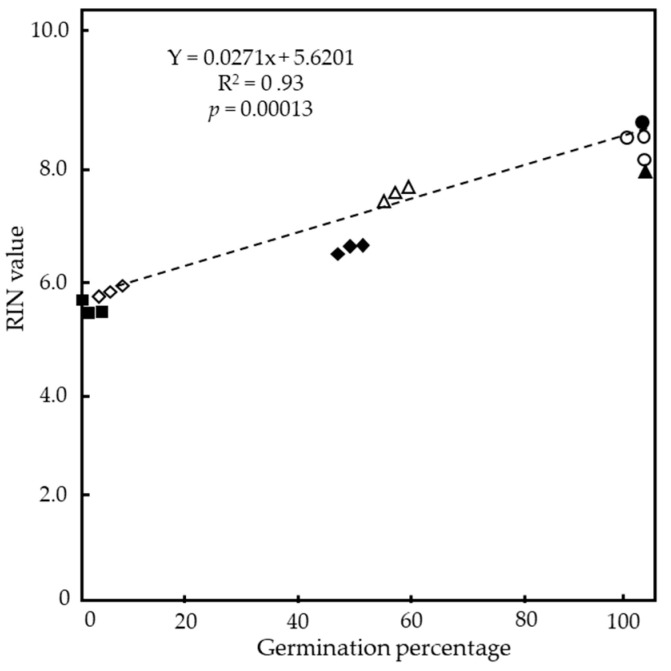
Relationship between germination percentages and RIN values of rice seeds in “top cultivars”, “middle cultivars”, and “low cultivars” of the NIAS world rice core collection exposed to CDT or stored at 4 °C for long years. RIN values are taken from Table 1, and germination data from Figure 2 and Figure 3 are used. Filled circle indicates without CDT (fresh seeds) for all (top–low) cultivars; open circles, triangles, and diamonds indicate top cultivars, middle cultivars, and low cultivars, respectively, after CDT. Filled triangle, diamonds, and squares indicate aged top cultivars, middle cultivars, and low cultivars, respectively. R^2^ = 0.93, and *p* = 0.00013.

To confirm the relationship between the longevity of seeds and RNA stability in the embryos, a correlation analysis between the germination percentages of the seeds and the mean RIN values of RNA extracted from the embryos of the seeds was performed. A scatter plot of the germination percentages and RIN values of the seeds after CDT and the aged seeds of the nine cultivars used in Figure 4 and Table 1 found a significant positive correlation between the germination percentage and RIN value (R^2^ = 0.93 at *p* = 0.00013) (Figure 5). 

## 3. Discussion

This study uncovers new insights into novel varietal differences in seed longevity within the NIAS world rice core collection (RDRS) following exposure to controlled deterioration treatment (CDT) under high temperatures and relative humidity. Among this world rice core collection, the seeds of three cultivars, “Vandaran (*indica*)”, “Tupa 729 (*japonica*)”, and “Badari Dhan (*indica*)”, which were grown in two cultivation seasons (2016 and 2019), consistently showed 100–80% germination even after long-term aging and under CDT. In addition, the long and stable longevity in the ”aged” seeds harvested in 2010 and 2013 of these three cultivars was confirmed in the experiments conducted under long-term storage at 4 °C. Conversely, “Urasan1”, “Jena 035”, and “Dianyu1” were particularly vulnerable and showed consistently near 0% germination after aging, low temperature, and CDT. The remaining cultivars showed a range of germination values from below 100% to 0% that varied between the conditions tested. This study clearly reveals that variations in germination percentages are found across varieties as a result of artificial or low-temperature aging conditions. This suggests that seed longevity is a heritable trait, implying that improvement in seed germinability through breeding could be achieved. Therefore, these three cultivars (“Vandaran”, “Tupa 729”, and “Badari Dhan”) could be used as valuable potential breeding materials for the improvement in seed longevity. Alternatively, these age-resistant cultivars may be crossed with the poor germination types to establish a segregating population to genetically define the important genomic regions or genes involved. In tomatoes, seed longevity was characterized by high heritability during growing at both optimal and high-temperature conditions and further identified heat-tolerant genotypes that could be used as breeding materials [43,44].

It has been shown that the RNA stability in dry seeds is involved in the conservation of seed germinability in various crops. In dry sunflower (*Helianthus annuus* L.) seeds and pea seeds, a strong correlation between seed germinability and the total RNA content was observed [35,36]. In the dry seeds of *Nicotiana* species, a correlation was found between seed germinability and the integrity of rRNA [37]. Finally, a loss of germination capacity by artificial aging treatment was accompanied by a reduction in the total RNA content and RNA integrity in the seeds of garden pea (*Pisum sativum*) and mung bean (*Vigna radiata* L.) [38,39]. Here, the analysis of embryonic RNA stability using RIN values revealed that the RNA integrity in the embryos of the seeds after CDT or long-term storage at 4 °C was significantly more intact in the cultivars with long seed longevity compared to the cultivars with short seed longevity. In the nine cultivars tested, classified by differences in seed longevity, a strong correlation (R^2^ = 0.93 at *p* = 0.00013) was detected between germination percentages and RIN values in the seeds with CDT and aged seeds. These results suggest that embryonic RNAs are an important part of seed germinability. We previously identified a high correlation between the degradation of embryonic RNAs and loss of seed germinability in three *japonica* rice cultivars, which further supports the importance of RNA stability for seed longevity [1] and the use of RIN values as a germinability index. A similar use of RIN values has been reported in the dry seeds of wheat and soybean crops where a significant correlation between germinabilities and RIN values was reported [45,46]. 

The data presented here, and in other studies, suggest that the stability of embryonic RNA integrity is significantly correlated to seed viability and longevity. The mechanism of RNA protection in age-resistant seeds that allows a seed to germinate after long periods of dormancy is an interesting area of research to identify the mechanisms of RNA maintenance over these long periods of time. Genetic segregation studies through crosses between the age-susceptible and age-resistant cultivars presented here may identify genes that are important towards this process. RNA-seq and RT-PCR experiments will help identify the RNAs that may be more prone to degradation over time compared to other more stable RNAs. The age-resistant rice cultivars identified here, which germinated despite long-term storage, low-temperature treatment, and CDT treatment, will provide important breeding material for the production of long-term stored rice seeds.

## 4. Materials and Methods

### 4.1. Seed Materials

Seeds of 69 rice (*Oryza sativa* L.) cultivars belonging to the world rice core collection developed by Kojima et al. [23] were obtained from the National Institute of Agrobiological Science (NIAS), Tsukuba, Ibaraki, Japan (Table 2). These seeds were cultivated and harvested during the rice-growing seasons (May to September) under natural conditions at the Toyama Agricultural, Forestry, and Fisheries Research Center in Toyama Prefecture, Toyama, Japan from 2010 to 2019. For the experiments, 44 to 56 cultivars with enough seeds were selected and used. The harvested seeds were stored at 4 °C and ~35% relative humidity until use. The seeds of these core collections, which were harvested in 2016 and 2019, were exposed to CDT after storage for one year and were used as “fresh seeds”. Seeds from the same collections were harvested in 2010 and 2013, respectively, and analyzed in 2020 as “aged seeds” in this study. 

### 4.2. Controlled Deterioration Treatment (CDT)

It takes several years to evaluate seed germinability under natural storage conditions. To circumvent this problem, an artificial aging approach known as controlled deterioration treatment (CDT) was employed to speed up the aging process with elevated temperatures and relative humidity for the quick estimation of seed viability [47,48]. In this report, seed samples were subjected to CDT for 40 days while being stored at 36 °C and 80% relative humidity in a sealed box with an open Petri dish containing saturated KCl solution as per our previous report [1]. In the meantime, aside from the usefulness of CDT as a quick vigor test, it has its limitations, which might not always fully mimic the natural seed aging treatment due to the substantial variability in aging conditions such as temperature, humidity, and duration [49,50,51]. Therefore, we next checked the germinability of aged seeds from the same rice core collections that were stored dry for 7 or 10 years. The provided data have been compared with the results obtained using seeds subjected to CDT [52,53,54].

### 4.3. Germination Assays

Germination tests were performed in triplicate with 50 seeds from each sample. The seeds were incubated for ten days in the dark in distilled water at 28 °C, with the water replaced every second day. Ten days after imbibition (DAI), the number of germinated seeds was counted. 

### 4.4. Extraction and Characterization of Total Embryonic RNAs

Dry embryos were separated from dehulled seeds using a surgical blade. Total RNA was extracted from 20 embryos using Fruit-mate for RNA purification and RNAiso Plus (Takara Bio Inc., Kusatsu, Shiga, Japan), according to the manufacturer’s protocol. The concentration and purity of extracted RNA was assessed at 260 and 280 nm using a NanoDrop 1000 spectrophotometer (Thermo Fisher Scientific Inc., Wilmington, DE, USA). The extracted total RNAs (200 ng) were analyzed by electrophoresis using an Agilent 2100 Bioanalyzer system (Agilent Technologies, Santa Clara, CA, USA, B.02.11); RIN values were then calculated by using the Agilent 2100 Bioanalyzer software (B.02.11). 

### 4.5. Statistical Analysis

The data underwent one-way analysis of variance (ANOVA), followed by Tukey’s Honestly Significant Difference (HSD) test to compare treatment means. JMP Pro 16 (JMP16.2, Cary, NC, USA) was used to conduct the statistical analysis. 

## Figures and Tables

**Table 1:** Comparison of mean RIN values in embryos of “Top, Middle, and Low cultivars” without or with CDT for 40 days and aged rice seeds.

**“Top Cultivars”**								
Vandaran			Tupa 729			Badari Dhan		
Fresh seeds	40 days CDT	Aged seeds	Fresh seeds	40 days CDT	Aged seeds	Fresh seeds	40 days CDT	Aged seeds
8.5 ± 0.4	8.4 ± 0.3	8.0 ± 0.1 **	8.5 ± 0.4	8.4 ± 0.4	8.0 ± 0.0 **	8.5 ± 0.4	8.4 ± 0.4	8.0 ± 0.1 **
**“Middle Cultivars”**								
Local Basmati			Hong Chew Zai			Davao 1		
Fresh seeds	40 days CDT	Aged seeds	Fresh seeds	40 days CDT	Aged seeds	Fresh seeds	40 days CDT	Aged seeds
8.5 ± 0.3	7.7 ± 0.4 **	6.7 ± 0.5 **	8.5 ± 0.4	7.5 ± 0.5 **	6.7 ± 0.3 **	8.5 ± 0.5	7.3 ± 0.3 **	6.6 ± 0.1 **
**“Low Cultivars”**								
Urasan 1			Jena 035			Dianyu 1		
Fresh seeds	40 days CDT	Aged seeds	Fresh seeds	40 days CDT	Aged seeds	Fresh seeds	40 days CDT	Aged seeds
8.5 ± 0.5	5.7 ± 0.2 **	5.6 ± 0.3 **	8.5 ± 0.3	5.8 ± 0.1 **	5.7 ± 0.1 **	8.5 ± 0.4	5.9 ± 0.3 **	5.6 ± 0.4 **

Values are the means of three replicates ± SE. Tukey’s test was used to compare the mean (n = 3) RIN values of the seeds for each cultivar “without CDT” (**: *p* < 0.01).

**Table 2:** List of NIAS world rice core collections used for evaluation of seed longevity in this study.

No.	ID	Cultivar Name	Type	Group ^(1)^	Origin
1	WRC 55	Tupa 729	*japonica*	A	Bangladesh
2	WRC 49	Padi Perak	*japonica*	A	Indonesia
3	WRC 45	Masho	*japonica*	A	Myanmar
4	WRC 22	Calatoc	*japonica*	A	Philippines
5	WRC 52	Khau Tan Chiem	*japonica*	A	Vietnam
6	WRC 50	Rexmont	*japonica*	A	USA
7	WRC 48	Khau Mac Kho	*japonica*	A	Vietnam
8	WRC 46	Khoa Nok	*japonica*	A	Laos
9	WRC 47	Jaguary	*japonica*	A	Brazil
10	WRC 01	Nipponbare	*japonica*	A	Japan
11	WRC 43	Dianyu 1	*japonica*	A	China
12	WRC 51	Urasan 1	*japonica*	A	Japan
13	WRC 53	Tima	*japonica*	A	Bhutan
14	WRC 35	ARC 5955	*indica-1*	B	India
15	WRC 41	Kaluheenati	*indica-1*	B	Sri Lanka
16	WRC 39	Badari Dhan	*indica-1*	B	Nepal
17	WRC 02	Kasalath	*indica-1*	B	India
18	WRC 33	Surjamukhi	*indica-1*	B	India
19	WRC 36	Ratul	*indica-1*	B	India
20	WRC 31	Shoni	*indica-1*	B	Bangladesh
21	WRC 34	ARC 7291	*indica-1*	B	India
22	WRC 37	ARC 7047	*indica-1*	B	India
23	WRC 29	Kalo Dhan	*indica-1*	B	Nepal
24	WRC 42	Local Basmati	*indica-1*	B	India
25	WRC 28	Jarjan	*indica-1*	B	Bhutan
26	WRC 32	Tupa 121-3	*indica-1*	B	Bangladesh
27	WRC 27	Nepal 8	*indica-1*	B	Nepal
28	WRC 25	Muha	*indica-1*	B	Indonesia
29	WRC 04	Jena 035	*indica-1*	B	Nepal
30	WRC 26	Jhona 2	*indica-1*	B	India
31	WRC 30	Anjana Dhan	*indica-1*	B	Nepal
32	WRC 38	ARC 11094	*indica-1*	B	India
33	WRC 40	Nepal 555	*indica-1*	B	India
34	WRC 16	Vary Futsi	*indica-2*	C	Madagascar
35	WRC 100	Vandaran	*indica-2*	C	Sri Lanka
36	WRC 03	Bei Khi	*indica-2*	C	Cambodia
37	WRC 44	Basilanon	*indica-2*	C	Philippines
38	WRC 57	Milyang23	*indica-2*	C	Korea
39	WRC 20	Tadukan	*indica-2*	C	Philippines
40	WRC 07	Davao 1	*indica-2*	C	Philippines
41	WRC 05	Naba	*indica-2*	C	India
42	WRC 17	Keiboba	*indica-2*	C	China
43	WRC 06	Puluik Arang	*indica-2*	C	Indonesia
44	WRC 99	Hong Cheuh Zai	*indica-2*	C	China
45	WRC 14	IR58	*indica-2*	C	Philippines
46	WRC 21	Shwe Nang Gyi	*indica-2*	C	Myanmar
47	WRC 13	Asu	*indica-2*	C	Bhutan
48	WRC 24	Pinulupot 1	*indica-2*	C	Philippines
49	WRC 23	Lebed	*indica-2*	C	Philippines
50	WRC 18	Qingyu Seiyu	*indica-2*	C	China
51	WRC 15	CO 13	*indica-2*	C	India
52	WRC 98	Deejaohualuo	*indica-2*	C	China
53	WRC 19	Deng Pao Zhai	*indica-2*	C	China
54	WRC 11	Jinguoyin	*indica-2*	C	China
55	WRC 09	Ryou Suisan Koumai	Others	D	China
56	WRC 10	Shuu Sou Shu	Others	D	China

^(1)^ Group “A” represents *japonica*-type cultivar, Group “B” represents *indica-1*, and Group “C” represents *indica-2* type cultivars, respectively [23,24].

## Data Availability

No new data were created or analyzed in this study.
